# Survival or Revival: Long-Term Preservation Induces a Reversible Viable but Non-Culturable State in Methane-Oxidizing Bacteria

**DOI:** 10.1371/journal.pone.0034196

**Published:** 2012-04-23

**Authors:** Sven Hoefman, Koenraad Van Hoorde, Nico Boon, Peter Vandamme, Paul De Vos, Kim Heylen

**Affiliations:** 1 Laboratory of Microbiology, Department of Biochemistry and Microbiology, Ghent University, Gent, Belgium; 2 Faculty of Applied Bioscience Engineering, University College Ghent, Gent, Belgium; 3 Laboratory of Microbial Ecology and Technology, Ghent University, Gent, Belgium; 4 BCCM/LMG Bacteria Collection, Gent, Belgium; Arizona State University, United States of America

## Abstract

Knowledge on long-term preservation of micro-organisms is limited and research in the field is scarce despite its importance for microbial biodiversity and biotechnological innovation. Preservation of fastidious organisms such as methane-oxidizing bacteria (MOB) has proven difficult. Most MOB do not survive lyophilization and only some can be cryopreserved successfully for short periods. A large-scale study was designed for a diverse set of MOB applying fifteen cryopreservation or lyophilization conditions. After three, six and twelve months of preservation, the viability (via live-dead flow cytometry) and culturability (via most-probable number analysis and plating) of the cells were assessed. All strains could be cryopreserved without a significant loss in culturability using 1% trehalose in 10-fold diluted TSB (TT) as preservation medium and 5% DMSO as cryoprotectant. Several other cryopreservation and lyophilization conditions, all of which involved the use of TT medium, also allowed successful preservation but showed a considerable loss in culturability. We demonstrate here that most of these non-culturables survived preservation according to viability assessment indicating that preservation induces a viable but non-culturable (VBNC) state in a significant fraction of cells. Since this state is reversible, these findings have major implications shifting the emphasis from survival to revival of cells in a preservation protocol. We showed that MOB cells could be significantly resuscitated from the VBNC state using the TT preservation medium.

## Introduction

Long-term preservation of micro-organisms is an often neglected but very important aspect of both applied and environmental microbiology [1], [2], [3]. Preservation of microbial resources allows (i) validation of previously obtained results, (ii) ensures that strains do not get lost during research, (ii) catalogs biodiversity for future research and (iv) enables scientists to apply well documented cultures for biotechnological or commercial use. Novel isolates can either be preserved in the research lab or deposited in public culture collections, safeguarding thousands of strains available for the scientific community upon request [4], [5]. Long-term storage is most often achieved by lyophilization or cryopreservation in liquid nitrogen or at −80°C [6]. The former requires specialized equipment and thus is mainly used by culture collections, and offers advantages over cryopreservation such as ease of storage and transport [7]. Besides the choice and concentration of cryo- or lyoprotectant, many other parameters may influence the success of preservation, such as the growth, preservation and resuscitation medium, growth rate, culture density, the rate of freezing and thawing or the instrument settings during lyophilization [7]. Therefore, long-term preservation is considered an empirical research field, where it is very difficult to compare or apply data from literature. Nonetheless, a wide variety of micro-organisms have already been successfully maintained for decades using these procedures [8]. However, standard preservation protocols appear to be less successful for many fastidious organisms, such as methane-oxidizing bacteria (MOB). Lyophilization of MOB is generally unsuccessful, while cryopreservation is possible but usually only for short periods [9], [10]. Therefore, many of these cultures can only be maintained by periodic sub-cultivation or are kept at low metabolic rates (4°C, under appropriate atmosphere). As a result, several MOB are no longer extant [11], despite being a well-studied and functionally important group of bacteria [12].

Upon resuscitation (after cryopreservation) or rehydration (after lyophilization), a preservation method is considered successful when strains are culturable (qualitatively) after preservation, preferably in high numbers (quantitatively), as evaluated by counting either most probable numbers (MPN) or colony forming units (CFU) [13]. Besides culturability, viability of cells can also be assessed based on certain cellular functions, such as membrane integrity [14]. For example, fluorescent dyes such as SYBR®Green can penetrate cells with intact cytoplasmic membranes, while propidium iodide only penetrates damaged membranes, resulting in green or red fluorescence respectively [15]. Cells in these different physiological states can then be counted rapidly by flow cytometry [16]. By applying such methods, it was discovered that cells can enter a state of dormancy [17], termed the viable but non-culturable (VBNC) state, at which cells are still alive but fail to grow using conditions that would normally allow their growth [18], [19]. The VBNC state is induced by certain stress factors, such as starvation, decreased temperatures or elevated osmotic concentrations [18], which are relevant for preserved cells, although the VBNC state has thus far not been linked to preservation. Considering that the VBNC state has been found to be reversible [20], it would be most informative to know whether a preservation method failed due to cell death or induction of the VBNC state, in order to plan resuscitation efforts accordingly.

The aim of the present study was to evaluate the success of long-term preservation of a diverse set of MOB by applying 15 preservation conditions using both lyophilization and cryopreservation. Viability was assessed through live/dead flow cytometry while culturability was evaluated through MPN analysis and plating. Since both viability and culturability were assessed quantitatively, it was explored whether non-culturable cells were either dead or still viable and whether the VBNC fraction could be reduced by resuscitation efforts. The obtained results showed that all MOB could be successfully preserved by cryopreservation and lyophilization, and demonstrated that a VBNC state induced by preservation could be significantly reduced using a specifically adapted preservation medium.

## Materials and Methods

### Strains, Growth Conditions and Preservation Cultures

Most of the aerobic methane-oxidizing diversity obtained so far is phylogenetically positioned within the *Alphaproteobacteria* (Type II MOB) and the *Gammaproteobacteria* (Type Ia/b MOB) [21]. Ten methane-oxidizing type strains were obtained from the DSMZ and NCIMB culture collections (www.dsmz.de and www.ncimb.com) and their main characteristics are listed in Table 1. Nine strains were cultivated in a diluted Nitrate Mineral Salts (dNMS) medium, as described previously [22], while DSM 19304^T^ was cultivated in DSM1180 medium (pH 9.0) (www.dsmz.de). Cultures were grown either in broth with 20% (v/v) CH_4_ added to the headspace or on solid medium in gas-tight jars (Oxoid, UK) under a CH_4_:air (1∶1) atmosphere.

**Table 1:** Preservation conditions with corresponding abbreviations (left) and MOB type strains with corresponding species name, type of MOB and standard cultivation conditions (right).

Preservation Condition^a^	Abbreviation	Strain	Species	Type	Standard medium	Temp. (°C)
**Lyophilization**		NCIMB 11130^T^	*Methylomonas methanica*	Ia	dNMS	28
20% Sucrose/10% BSA	S/BSA	DSM 13736^T^	*Methylosarcina fibrata*	Ia	dNMS	28
20% Sucrose/10% BSA (TT)	S/BSA/TT	DSM 19304^T^	*Methylomicrobium alcaliphilum*	Ia	DSM1180	28
7.5% Trehalose in horse serum	T/HS	NCIMB 11912^T^	*Methylocaldum gracile*	Ib	dNMS	28
7.5% Trehalose in horse serum (TT)	T/HS/TT	NCIMB 11853^T^	*Methylococcus capsulatus*	Ib	dNMS	37
10% Skimmed Milk	S.Milk	DSM 17706^T^	*Methylosinus sporium*	II	dNMS	28
12% Glycine Betaine	LPA-GB	NCIMB 11131^T^	*Methylosinus trichosporium*	II	dNMS	28
**Cryopreservation (liquid** **nitrogen)**		DSM 18500^T^	*Methylocystis hirsuta*	II	dNMS	28
15% Glycerol	Glyc	NCIMB 11129^T^	*Methylocystis parvus*	II	dNMS	28
15% Glycerol (TT)	Glyc/TT	DSM 15673^T^	*Methylocella tundrae*	II	dNMS	20
5% DMSO	DMSO					
5% DMSO (TT)	DMSO/TT					
5% DMSO^b^	DMSO/−80					
10% Methanol	Methanol					
20% Sucrose	Sucrose					
12% Glycine Betaine	CPA-GB					
Microbank Beads^b^	Beads					

Use of TT preservation medium for growth, preservation and resuscitation of cells is mentioned in brackets. Standard medium was used for the other preservation conditions. DSM 19304^T^ could not be cultivated in TT medium.

a Final concentrations are listed.

b Conditions preserved at −80°C and not in liquid nitrogen.

Prior to preservation, the MOB strains were grown in broth at optimal temperature (Table 1) to early stationary phase in standard growth medium (dNMS or DSM1180) as well as in a carbon-rich preservation medium, i.e. a ten-fold diluted trypticase soy broth (BD, France) medium supplemented with 1% trehalose (TT medium). DSM 19304T could only be cultivated in DSM1180 medium. To allow harvesting of cells at early stationary phase, growth curves for all cultures and in each medium were established in preliminary experiments (data not shown).

### MPN and Plate Enumeration

To enumerate the culturable MOB at early stationary phase, either most probable number (MPN) or plate counts were performed. For MPN counts, a dilution series (10^−2^ up to 10^−9^) was prepared in triplicate in 96-well microtiter plates (200 µL/well) and incubated in dNMS or DSM1180 medium at optimal temperature (Table 1) in gas-tight jars (Oxoid, UK) under a CH_4_:air (1∶1) atmosphere. After one and two weeks of incubation, growth was monitored by measuring the optical density at 600 nm (Spectramax+384, Molecular Devices, USA) and most probable numbers were calculated using MPN-Tables [23]. Plate counts were estimated by plating dilutions 10^−1^, 10^−3^ and 10^−5^ in duplicate (50 µL/plate) on dNMS or DSM1180 medium and incubated as mentioned higher. The amount of colony forming units per volume (CFU/mL) was estimated after one and two weeks of incubation. For logistic reasons, culturability was quantified by MPN for the eight strains grown at 28°C, and by plate counting for the remaining two strains.

### Viability Counts by Flow Cytometry

MOB were quantified by live/dead flow cytometry (FCM). The cultures were diluted to optimal cell density (10^4^–10^6^ cells/mL) for FCM measurements and 10 µL mL^−1^ live/dead stain, 5 mM Na_2_EDTA and 10 µL mL^−1^ Cytocount^™^ beads at 1,064 particles µL^−1^ (Dako, Denmark) were added. The live/dead stain was composed of 30 mM propidium iodide (Invitrogen Switzerland) mixed with SYBR®Green I (10^−2^ dilution in dimethyl sulfoxide (DMSO)) at a ratio of 1∶50. Following staining, samples were mixed thoroughly and incubated in the dark for 15 min at room temperature. Samples were analyzed using a CyAn™ High Performance Flow Cytometer (Dakocytomation, Belgium) equipped with a 50-mW sapphire solid-state diode laser, emitting at a fixed wavelength of 488 nm. The optimal settings for each strain were determined as described by [15]. Briefly, the instrument voltage values for side angle light scatter (SSC), green fluorescence and red fluorescence were optimized for log-phase MOB cells. No compensation was applied to sample analysis. For each sample run, log-phase cells of stained MOB strains and non-stained MOB strains were used as positive and negative controls, respectively. Heat-killed (90°C for 3 min) cells were used as inactivation control for the stains applied in the flow cytometric analysis. Samples were run until a bead count of approximately 300 was reached at an EPS (Events Per Second) rate below 10,000/s. Green fluorescence was collected at 510−550 nm, red fluorescence at 603−623 nm. Data were analyzed using Summit™ 4.3 software. Following the principle that only SYBR® Green I penetrates cells with intact membranes resulting in green fluorescence, and both SYBR®Green I and propidium iodide penetrate cells with destroyed membranes resulting in red fluorescence, the amount of viable cells mL^−1^ (based on bead counts) of the MOB cultures was calculated.

### Preservation

#### (i) Lyophilization

Ten MOB type strains were lyophilized. Samples were centrifuged for 15 min at 6,000 g and resuspended in a lyoprotectant (LPA). The six LPA combinations are listed in Table 1. For conditions in TT medium, besides the preservation of cultures in this medium in combination with an LPA, cultures were also grown prior to preservation and rehydrated after preservation in TT medium. For clarity, TT medium will be further termed as a ‘preservation medium’, although this is a simplification for ‘pre-preservation growth, preservation and rehydration medium’. The combination of cultures with protectants resulted in 58 suspensions. Ampoules (AR-glass, 7 mm diameter, 0.9 mm thickness) were filled with 100 µL of each suspension in triplicate (one for each evaluation time-point), a cotton plug was implemented and the suspensions were lyophilized: after an initial freezing step at −50°C for 60 min in a pre-cooled plate (−50°C), the ampoules were subjected to a primary (−18°C, 0.5 mbar, 410 min) and secondary drying phase (20°C, 0.012 mbar, 746 min). Ampoules were subsequently heat-sealed under vacuum (<0.13 mbar) and stored at 4°C in the dark.

#### (ii) Cryopreservation

The ten MOB type strains cultivated as explained above were subjected to long-term preservation by cryopreservation in liquid nitrogen. Cryotube^™^ vials (Nunc, Denmark) were used to add 500 µL sample to 500 µL cryoprotectant (CPA). The eight CPA combinations are listed in Table 1. As mentioned above, for conditions in TT medium, this medium was used as a pre-preservation growth, preservation and resuscitation medium, but is further termed ‘preservation medium’ for clarity. Vials were stored in liquid nitrogen, in the gas phase just above the liquid phase. DMSO as a CPA was also tested at −80°C. The combination of cultures with protectants resulted in 78 suspensions, prepared in triplicate (one for each evaluation time-point). When glycerol, sucrose and betaine were used as a CPA, contact between cells and CPA was at least 60 min to allow cellular uptake of CPA prior to preservation. Manipulations with DMSO and methanol in contact with cells were executed at 4°C and care was taken to preserve the cells immediately after addition of these CPA, to avoid toxic effects. The MOB were also cryopreserved using Microbank^™^ beads (Pro-Lab Diagnostics, USA). After centrifuging (15 min, 6,000 g) 1 mL of each culture grown in carbon-deficient media, the cell pellet was harvested using sterile cotton swabs and beads were inoculated with cell material according to the manufacturer’s instructions. The vials were preserved at −80°C.

### Rehydration and Resuscitation

The lyophilized suspensions were rehydrated after 3, 6 and 12 months. Ampoules were opened and 1 mL fresh medium (either dNMS, DSM1180 or TT medium) was added to rehydrate the cells and the suspensions were transferred to eppendorf tubes. After a rehydration step at room temperature for approximately one hour, the culturable and viable recovery of the cells was evaluated as above.

The cryopreserved suspensions were resuscitated after 3, 6 and 12 months. Vials were thawed at 37°C, and upon thawing, immediately transferred to eppendorf tubes and centrifuged (6,000 g, 15 min). After supernatant removal, an equal volume of fresh medium (either dNMS, DSM1180 or TT medium) was added to avoid toxic effects of DMSO and methanol at room temperature. After a resuscitation step at room temperature for approximately one hour, the culturable and viable recovery of the cells was evaluated as above. The survival of the cultures on Microbank^™^ beads was evaluated on plates according to the manufacturer’s instructions. Since quantification is not possible using the beads, culturability was only assessed qualitatively for this preservation condition.

### Quality Control

Prior to preservation, the ten MOB type strains were verified for absence of heterotrophic satellites on trypticase soy agar after 2 weeks of incubation without any additional CH_4_ in the atmosphere. In addition, 16S rRNA gene sequence analysis was performed on the cultures, as described previously [24]. Both quality control tests were also performed on the cryopreserved cultures after a 12-month preservation period with DMSO as CPA in standard medium as well as in TT medium.

### Statistical Analysis

Prior to statistical analysis, recorded measurements were log transformed. Imputation of missing values was performed by implementing the expectation-maximization (EM) maximum likelihood estimation (MLE) algorithm, with the maximum number of iterations set at 1000. Nested analysis of variance (ANOVA) designs with repeated measures were used for evaluating the possible effect of the duration of preservation and the effect of LPA or CPA conditions used on culturability and viability measured by MPN and FCM, respectively. Depending on whether or not the assumption of sphericity was respected, no correction (Mauchly’s test of sphericity, p>0,05) or a Greenhouse-Geisser or Huynh-Feldt correction (Mauchly’s test of sphericity, p<0,05 with estimated epsilon value ε<0,75 or ε<0,75 respectively) was taken into account. ANOVA implementing Scheffé’s post-hoc test was used to assess which preservation condition scored significantly better than others. The effect of adding TT was assessed using a paired samples T-test. All statistical data analyses were performed using IBM SPSS Statistics 19 (IBM, Brussels, Belgium).

## Results

### Dataset Generation for Long-term Preservation of MOB

**Figure 1:**
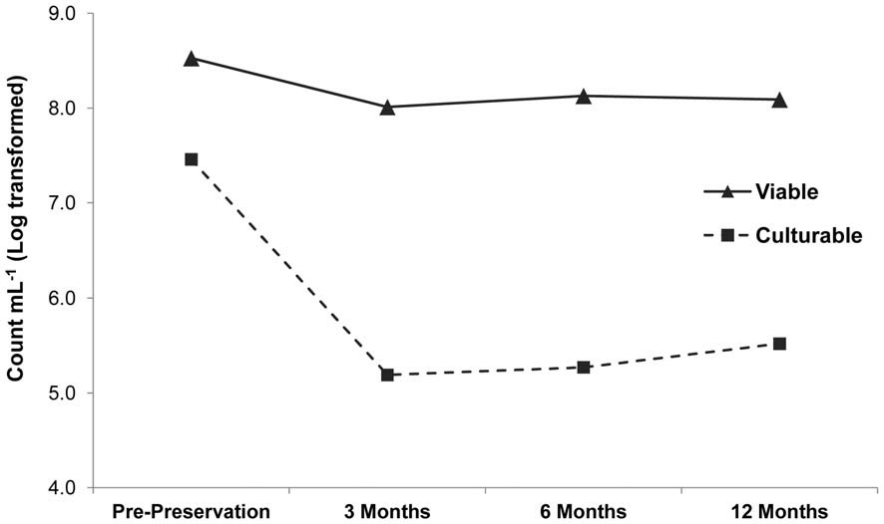
Effect of long-term preservation on MOB viability and culturability after 3, 6 and 12 months. The average viable (black triangle) and culturable (black square) log transformed cells per mL of all tested strains in all preservation conditions was plotted over time: before preservation & 3, 6 and 12 months post-preservation. For both the viable as well as the culturable counts, counts were significantly lower after preservation (p-value<0.05), while the 3 tested time points post-preservation were similar compared to one another (p-value>0.05).

Currently, there is no universal protocol for the long-term preservation of methane-oxidizing bacteria. Therefore, a diverse set of ten type strains of MOB, representing eight genera and the three major MOB types (Type Ia, Ib & II), was tested using 15 preservation conditions (Table 1). The conditions were evaluated after 3, 6 and 12 months of preservation, either by cryopreservation or lyophilization. The success rate of different preservation conditions can be evaluated by simply checking if resuscitated cultures can still grow, or more thoroughly, by counting the fraction of cells that are viable (based on FCM) and the fraction that can grow (based on MPN and plating). As a result of cell death due to the harsh conditions during preservation, it is expected that both these fractions will drop. Indeed, Figure 1 displays the average log transformed outcome of all combinations of cultures and preservation conditions and shows a significant drop (p-value <0.05) in both viability and culturability after preservation. Interestingly, the three tested time points post-preservation were found to be highly similar, indicating that there was no decline (p-value <0.05) in viability or culturability over the tested time frame. Therefore, the data obtained after 12-month preservation were selected to investigate differences between preservation conditions and counts more deeply.

### Qualitative Evaluation of MOB Culturability

For each condition, the percentage of cultures that could still grow after 12 months preservation was calculated (Table 2). Three conditions were found to preserve all strains successfully: DMSO/TT (cryopreservation), Glyc/TT (cryopreservation) and S/BSA/TT (lyophilization). Interestingly, in these three preservation conditions TT medium was used instead of standard growth medium as pre-preservation growth, preservation and resuscitation medium and performed better than their corresponding condition in standard medium, even when excluding results of DSM 19304^T^, which was not tested in TT medium (Table 2). The lowest culturability rate (10%) was observed with glycine betaine as a lyoprotectant, while most of the other preservation conditions resulted in culturability rates around 60−90%. NCIMB 11912^T^ appeared to be the most difficult strain to grow after preservation with overall culturability of 47%, while this ranged between 60−93% for the other strains. Strain authenticity was confirmed by 16S rRNA gene sequence analysis and absence of growth on TSA plates.

**Table 2:** The percentage of strains that were culturable or viable per preservation condition (left) and the percentage of preservation conditions for which culturability or viability was achieved per strain (right) after 12 months of preservation.

Condition	Culturable (%)	Viable (%)	Type Strain	Culturable (%)	Viable (%)
**Lyophilization**			DSM 18500^T^	87	93
S/BSA	80 (78)	80 (90)	DSM 13736^T^	87	64
S/BSA/TT	100	100	DSM 17706^T^	93	93
T/HS	60 (67)	100 (100)	DSM 15673^T^	87	100
T/HS/TT	89	100	NCIMB 11912^T^	47	92
S.Milk	70	83	NCIMB 11130^T^	73	100
LPA-GB	10	44	NCIMB 11129^T^	93	100
**Cryopreservation**			NCIMB 11131^T^	93	100
Glyc	80 (78)	100 (100)	NCIMB 11853^T^	60	93
Glyc/TT	100	100	DSM 19304^T^	64	89
DMSO	90 (89)	100 (100)			
DMSO/TT	100	100			
DMSO/−80	90	100			
Methanol	80	90			
Sucrose	80	90			
CPA-GB	90	100			
Beads	60	NT			

Values between brackets exclude DSM 19304^T^, the strain not tested in TT medium, to allow a more accurate comparison of the four conditions tested in TT medium with their corresponding conditions in standard medium.

### Quantitative Evaluation of MOB Culturability

To investigate the success of each condition in more detail, the culturability was examined quantitatively: a condition is more successful when the drop in culturability after preservation is lower. Based on the average culturability log transformed drops, a ranking of the preservation conditions was calculated (Table 3). Interestingly, the condition DMSO/TT, which allowed preservation of all strains successfully based on qualitative data (Table 2), also resulted in the lowest culturability drops quantitatively. This suggests that MOB can be stored most successfully through cryopreservation in liquid nitrogen with 5% DMSO using TT preservation medium. Four out of six lyophilization conditions were less successful than most cryopresentation conditions (group B&C, Table 3). However, the other two lyophilization conditions, S/BSA/TT and T/HS/TT, notably the only ones using the TT medium, were grouped among the best conditions (group A, Table 3). Considering that S/BSA/TT was one of the three conditions suited for successful preservation of all strains, lyophilization of MOB combining sucrose and BSA as LPA in TT as preservation medium can be considered as a formidable alternative to cryopreservation. The preservation condition using glycerol, the most common CPA, was found among the worst preservation conditions of this study. However, glycerol scored markedly better in combination with TT medium, similar as was observed for the other preservation conditions using TT medium. No significant difference was observed when comparing the preservation of DMSO in liquid nitrogen or at −80°C. Lyophilization of MOB using glycine betaine was the least successful condition in the present study, both qualitatively (Table 2) and quantitatively (Table 3).

**Table 3:** Ranking and separation into homogenous subsets using Scheffé’s test of tested preservation conditions based on average log transformed culturability data.

Ranking	Condition	Method	Group (Scheffé’s testa,b)		
			A	B	C
1	DMSO/TT	Cryopreservation	0.0^c^		
2	DMSO	Cryopreservation	0.7		
3	Glyc/TT	Cryopreservation	0.8		
4	CPA-GB	Cryopreservation	1.0		
5	DMSO/−80	Cryopreservation	1.0		
6	Methanol	Cryopreservation	1.2		
7	T/HS/TT	Lyophilization	1.3		
8	Sucrose	Cryopreservation	1.5		
9	S/BSA/TT	Lyophilization	1.7		
10	Glyc	Cryopreservation		2.0	
11	S/BSA	Lyophilization		2.1	
12	T/HS	Lyophilization		3.2	
13	S.Milk	Lyophilization		3.4	
14	LPA-GB	Lyophilization			5.3

a Groups A & B and B & C are homogenous subsets (no significant difference). Members of group A are significantly different from members of group C

(p-value<0.05).

b Average mean culturable log drop (compensated for missing values).

c Increasing culturable log drops from top to bottom.

### VBNC State Induced by Long-term Preservation of MOB

**Figure 2:**
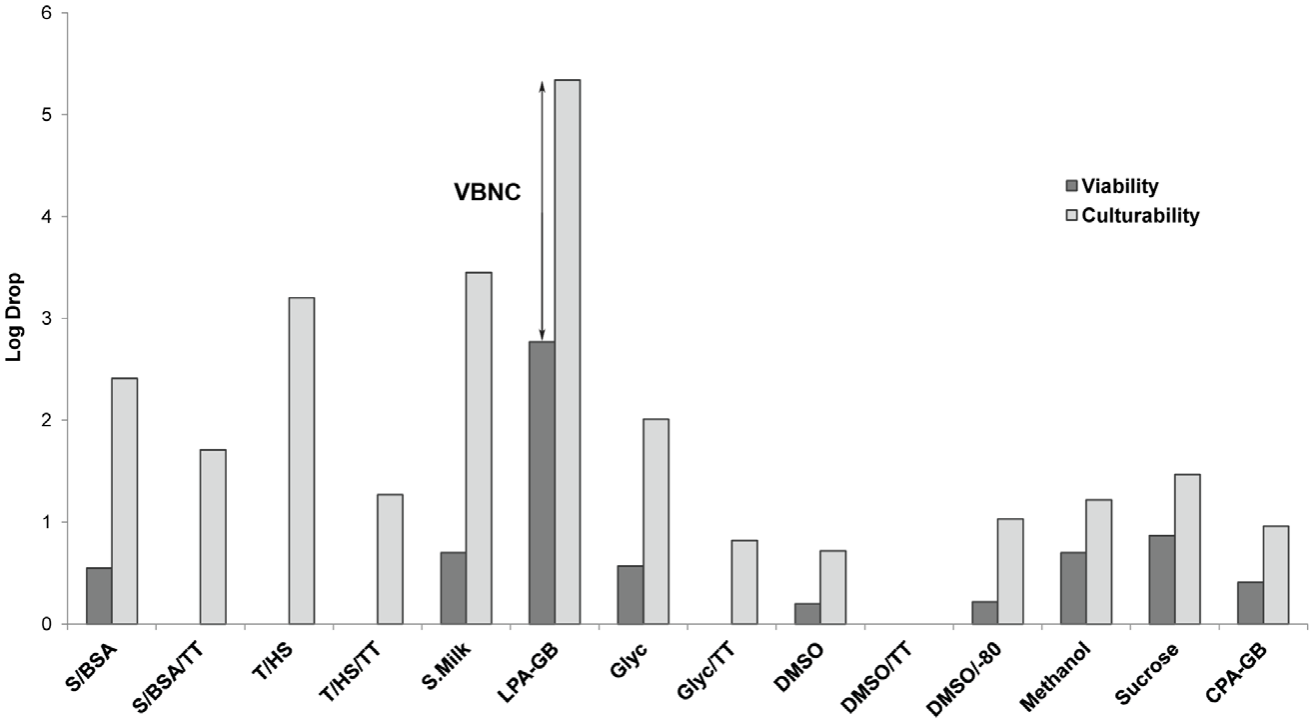
Average viable (dark grey bars) and culturable (light grey bars) log drops of all tested strains plotted for each preservation condition. Log drops were obtained by subtraction of 12 month post-preservation counts by pre-preservation counts using log transformed data. Following preservation, the drop in culturability is mostly more extensive (larger bars) than the drop in viability indicating that a vast number of cells became viable-but-non-culturable (VBNC) after the preservation process (arrow indicating VBNC as difference between viable and culturable log drop for condition LPA-GB). Bars that are not visible indicate that no drop was observed after preservation (e.g. DMSO/TT for both viable and culturable log drop counts). The use of TT medium before, during and after preservation reduced the amount of VBNC cells significantly (p-value<0.05; e.g. T/HS/TT compared with T/HS) in a comparison between the four conditions using TT with the four corresponding conditions in standard medium. When the viability drops, the culturability also drops but to a higher extent, it may indicate that FCM can be used to partially predict the success of subsequent cultivation attempts following long-term preservation.

Besides data on culturability, Table 2 also lists the percentage of strains that were still viable after 12 months preservation, according to flow cytometric live-dead counts. Qualitatively, nine preservation conditions resulted in 100% viability of the tested strains, while this was only true for three preservation conditions based on culturability (Table 2). Quantitatively, the drop in culturability was found to be significantly larger than the drop in viability (p-value<0.05), as visualized for the total dataset (Figure 1) as well as for each preservation condition (Figure 2) and each strain per condition (Table S1). Indeed, Figure 2 shows that the average culturable drop is more extensive (larger bars) than the average viable drop. These results indicate that in addition to a fraction of cells that die off during preservation, a viable but non-culturable (VBNC) state is also induced. The VBNC state was defined here as the increased difference between viable FCM counts and culturable counts induced by preservation (arrow Figure 2), while resuscitation from the VBNC state was assumed when this difference could be reduced. To evaluate the effect of TT medium on the VBNC fraction, a separate dataset was created excluding DSM 19304^T^ and including only the four preservation conditions with TT medium and their corresponding conditions in standard medium. Statistical analysis showed that the fraction of viable cells was similar for both media. However, for the TT medium a significantly higher culturable fraction was observed, or in other words, the fraction of VBNC cells was significantly reduced by use of TT medium.

**Figure 3:**
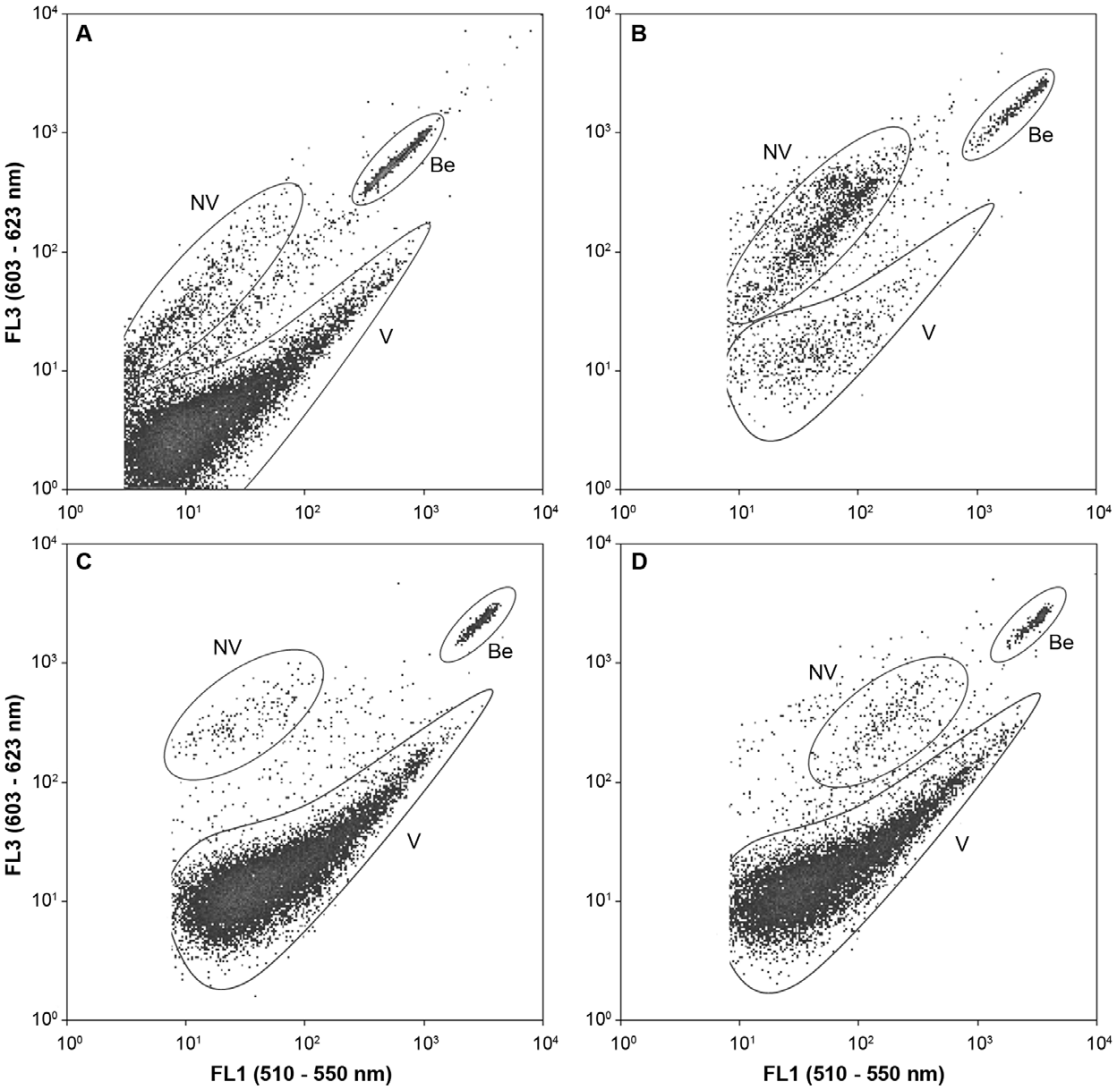
Live-dead staining of *Methylocystis parvus* NCIMB 11129^T^ prior to preservation (A) and preserved for 12 months using LPA-GB (B), glycerol (C) and DMSO/TT (D) measured by flow cytometry. Green fluorescence (FL1 detector) was plotted against red fluorescence (FL3 detector). Cells emitting more green than red were considered viable (V), cells emitting more red than green were considered non-viable (NV). Cytocount beads (Be) were added to calculate the amount of viable cells per mL. These types of counts were encircled for indicative purposes only. Background counts found at the bottom left are not shown for clarity. Since obtained non-viable counts were expected to be underestimated (detritus of cells causing increase in background), these were not used in actual calculations. Before preservation (A) the majority of cells were viable. However, lyophilization of NCIMB 11129^T^ with LPA-GB (B) killed the majority of cells (shift from viable to non-viable), which corresponded with an MPN count below detection limit (Table S1). In contrast, a drop in viability could not be observed between pre-preservation of NCIMB 11129^T^ and post-preservation using glycerol (C) and DMSO/TT (D). Interestingly, despite their similar flow cytometric counts and profiles, the culturable fraction was a lot higher after preserving with DMSO/TT (MPN log drop 0.7; Table S1) than with glycerol (MPN log drop 2.6; Table S1) indicating that the VBNC fraction was limited in the former and extensive in the latter condition.

As a detailed example, the effect of long-term preservation on the viability of strain NCIMB 11129^T^ is shown in Figure 3. Prior to preservation most of the cells of the culture were considered viable by visual inspection, due to a larger emission of green fluorescence (SYBR®Green dye) than red fluorescence (PI dye). The majority of the cells was no longer viable after preserving this culture with LPA-GB (B, Figure 3), which corresponded to an expected large drop in culturability (below detection limit, Table S1). Since the FCM profiles and counts after preservation with either Glyc or DMSO/TT were highly similar to the profile and counts prior to preservation, similar cultivation counts were expected. Interestingly, the drop in culturability was a lot higher for Glyc (2.6 logs) than for DMSO/TT (0.7 logs), suggesting that VBNC was induced due to preservation but could be reduced depending on the protectant or preservation medium used. Such FCM profiles were obtained and processed for all combinations of strains and preservation conditions for each time-point (data not shown). FCM profiles of preserved samples showed a large increase in background, which was probably caused by detritus of cells destroyed during the preservation process. Therefore, obtained non-viable counts were probably underestimated and were not used in further calculations.

The drops in viability and culturability as well as the VBNC cell fraction caused by preservation (calculated as the drop in culturability subtracted by the drop in viability) per preservation condition for each of the strains are shown in Table S1. When a clear drop in viability was observed (e.g. for conditions S/BSA, S.Milk and LPA-GB), a corresponding more extensive drop in culturability was observed (Figure 3). The opposite was not necessarily so, since for S/BSA/TT the large drop in culturability did not correspond with a clear drop in viability.

## Discussion

The essence of preserving biomass is the possibility to again grow cells when needed. Therefore, cultivation is the routine evaluation tool of preservation success. Unfortunately, this approach does not allow differentiation between cell death and survival of cells unable to replicate in given cultivation conditions (i.e. VBNC cells). The VBNC state is known to be induced by stress but has thus far never been linked to preservation. Here, we unequivocally demonstrated that recovery of culturability and not viability of biomass after preservation is most problematic: a significant discrepancy between viability and culturability after preservation was observed for ten MOB type strains (representing the three major types of aerobic MOB; Type Ia, Ib & II) in fifteen different preservation conditions analyzed in parallel by live/dead flow cytometry and cultivation, respectively. To overcome possible subjective and difficult interpretation of FCM data (as described by [25], [26]), the general trend of high viability was confirmed by highly reproducible results between time points and the observation of an extensive viability drop for the worst preservation conditions. In addition, viable counts with FCM and MPN were highly similar for easily preserved strains *Bacillus cereus* LMG 6923^T^ and *Pseudomonas aeruginosa* LMG 1242^T^ that do not demonstrate VBNC due to their high recovery of culturability (data not shown). Injured cells mimicking the VBNC state, and thus not truly dormant cells [27], [28], were not expected in our dataset since shifts towards increased red fluorescence were not apparent (Figure 3), although this cannot be completely excluded without conclusive tests [18], [25]. However, regardless of whether cells are damaged or dormant after preservation, viability assessment proved a valuable asset to preservation methodology for partial prediction of cultivation success (as biomass with high viability drops also showed low culturability). This is of particular interest for slow-growing fastidious micro-organisms, since the rapidly obtained FCM results could be used to focus subsequent laborious cultivation attempts only on biomass with a high viable fraction. Alternatively, culture collections could use FCM as a quick tool as part of their quality control.

Preservation induced a viable but non-culturable state in a significant amount of cells. Since this state can be reversible [20], additional efforts to resuscitate preserved organisms become crucial aspects of the preservation methodology. Interestingly, the innate cryoprotective effects of carbon-rich growth media is often overseen [8], [29], because to date most preservation studies focused on heterotrophic bacteria, but it could possibly explain the ease of preserving this kind of microorganisms. Indeed, use of TT medium containing ten-fold diluted TSB and trehalose (which protects cells against desiccation effects during lyophilization and rehydration [30], [31]) as preservation medium in the present study did significantly reduce the fraction of VBNC cells of MOB strains, which are normally cultivated in carbon-deficient media [10]. Current data does not allow deduction of the specific cause of this effect, which could be the use of a carbon-rich medium during growth (cellular uptake of protective compounds), preservation or resuscitation, the specific use of TSB and trehalose for this medium, its combination with an extra cryo- and lyoprotectant, or a combination of the above. One can argue that, as preservation is an empirical research field, it is not vital to know the exact mechanisms of the protective role as long as resuscitation improves. Nevertheless extrapolation of our findings to other bacterial groups requires extra tests to pinpoint the resuscitation effect to a specific cause.

In addition to a suitable medium choice, there are alternative ways to resuscitate VBNC cells, such as extended incubation [32], a temperature shift, addition of peptidoglycan hydrolases [18], [19] or signal molecules such as N-acyl homoserine lactones or short peptides [33], [34] to the cultivation medium. Filter-sterilized supernatants of fresh cultures are hypothesized to produce resuscitation promoting factors [35] which can increase culturability through quorum sensing [36]. Unfortunately, tests with filter-sterilized spent medium were unsuccessful here (data not shown). On the other hand, a five-week prolonged MPN analysis did indeed increase culturability for 50% of the tested cases (combination of strains with protectants; data not shown). These observations were unexpected since culturability results after one and two weeks were similar to prior preservation, but could be explained by the scout model proposed by Epstein [37], [38]. This model describes that a small fraction of VBNC cells can ‘wake up’ stochastically and start a new population. In this way, the culturable fraction has a growth rate similar to the culture prior to preservation, while scouts from the VBNC fraction create additional positive wells upon extended incubation.

Preservation of methane-oxidizing bacteria is often reported as problematic and limited in time [10], but little comparable information is available. By combining fifteen cryopreservation or lyophilization conditions, ten MOB type strains were successfully preserved for twelve months. Only when using 5% DMSO in combination with a 1% trehalose in ten-fold diluted TSB (TT) preservation medium, all strains could be resuscitated after cryopreservation in liquid nitrogen without a significant quantitative drop in culturability. However, two other conditions could also preserve all strains, namely cryopreservation with glycerol in TT medium and lyophilization with sucrose and BSA in TT medium. Lyophilization was previously deemed unsuccessful for MOB and advised against [9] but has several advantages over cryopreservation, such as ease of storage and distribution of the material [7]. In absence of facilities for liquid nitrogen storage or lyophilization, laboratories can still successfully cryopreserve MOB at −80°C, as was shown in a comparison with DMSO as CPA.

Even without use of TT medium and contradictory to previous reports, cryopreservation with standard CPA’s such as DMSO, glycerol and methanol allowed preservation of most strains, without decline over time (no differences in culturability and viability were observed between the datasets after three, six and twelve months of preservation). The presented results indicate that by applying appropriate preservation strategies as also discussed thoroughly by Tindall [39] and Adams [40], a higher success rate could be achieved using similar conditions as previously reported. Since cell damage is mainly inflicted during freezing and thawing events and not during storage [41], [42], a decline in viability or culturability is highly unlikely within testable time frames [43], if cells are stored at stable low temperatures such as in liquid nitrogen containers. Furthermore, as performed in the present study, protectant exposure time should be adapted based on each type of protectant to improve preservation and resuscitation efficiency or to limit toxicity [44].

In conclusion, the present study demonstrated that recovery of culturability and not viability is the bottleneck for reviving preserved MOB. Therefore, improved resuscitation procedures should become a key aspect of preservation protocols. It was shown here that the use of a combination of TSB and trehalose in the growth, preservation and resuscitation medium with 5% DMSO as cryoprotective agent significantly reduced the induced viable but non-culturable state. Finally, this was further confirmed by successfully preserving 22 recently isolated MOB [24], twenty of which were type I MOB reported to be the most difficult to preserve [10], as well as eight ammonia-oxidizing bacteria (functionally highly related to MOB) from all four AOB genera [45] (data not shown). Therefore, the here applied methodology is most probably more widely applicable for fastidious organisms.

## Supporting Information

Table S1 **The log drop in viability and culturability and the VBNC fraction for each strain tested with each preservation condition.** The VBNC fraction was calculated by subtraction of the culturable drop by the viable drop. Detection limit values were used to calculate drops with counts that were below this limit, such calculations are marked with ≥, indicating that the true drop is potentially even higher. Calculations of drops that resulted in negative values were indicated as 0, since these would implicate an impossible increase after preservation, attributed to variations in the measurements.
(DOC)
