# Supplementary material for: Survival or Revival: Long-Term Preservation Induces a Reversible Viable but Non-Culturable State in Methane-Oxidizing Bacteria
Source: PLoS One. 2012 Apr 23;7(4):e34196. doi: 10.1371/journal.pone.0034196 (PMC3335116; doi:10.1371/journal.pone.0034196)
Supplement: Table S1 — The log drop in viability and culturability and the VBNC fraction for each strain tested with each preservation condition. The VBNC fraction was calculated by subtraction of the culturable drop by the viable drop. Detection limit values were used to calculate drops with counts that were below this limit, such calculations are marked with ≥, indicating that the true drop is potentially even higher. Calculations of drops that resulted in negative values were indicated as 0, since these would implicate an impossible increase after preservation, attributed to variations in the measurements. (DOC) [file pone.0034196.s001.doc]

**Table S1: The log drop in viability and culturability and the VBNC fraction for each strain tested with each preservation condition.** The VBNC fraction was calculated by subtraction of the culturable drop by the viable drop. Detection limit values were used to calculate drops with counts that were below this limit, such calculations are marked with ≥, indicating that the true drop is potentially even higher. Calculations of drops that resulted in negative values were indicated as 0, since these would implicate an impossible increase after preservation, attributed to variations in the measurements.

| **Preservation Condition^a,b^** | | | | | | | | | | | | | | | | | | | | | | | | | | | | | |
| --- | --- | --- | --- | --- | --- | --- | --- | --- | --- | --- | --- | --- | --- | --- | --- | --- | --- | --- | --- | --- | --- | --- | --- | --- | --- | --- | --- | --- | --- |
| **Data Processed** | **Type Strain** | **Lyophilization** | | | | | | | | | | | | **Cryopreservation** | | | | | | | | | | | | | | | |
|  |  | S/BSA | | S/BSA/TT | | T/HS | | T/HS/TT | | S.Milk | | LPA-GB | | Glyc | | Glyc/TT | | DMSO | | DMSO/TT | | DMSO/-80 | | Methanol | | Sucrose | | CPA-GB | |
| Viable Drop | DSM 18500^T^ |  | 0.0 |  | 0.0 |  | ND |  | 0.5 |  | 0.0 | ≥ | 4.6 |  | 0.0 |  | 0.0 |  | 0.0 |  | 0.0 |  | 0.0 |  | 0.0 |  | 1.0 |  | 0.0 |
|  | DSM 13736^T^ | ≥ | 3.8 |  | 0.0 |  | 0.0 |  | 0.0 | ≥ | 2.6 | ≥ | 4.0 |  | 1.2 |  | 0.0 |  | 0.8 |  | 0.0 |  | 0.0 | ≥ | 4.0 | ≥ | 3.9 |  | 1.6 |
|  | DSM 17706^T^ |  | 0.0 |  | 0.0 |  | 0.8 |  | 0.0 |  | 0.0 | ≥ | 4.2 |  | 0.1 |  | 0.0 |  | 0.0 |  | 0.0 |  | 1.4 |  | 0.0 |  | 0.0 |  | 0.0 |
|  | DSM 15673^T^ |  | 0.0 |  | 0.0 |  | 0.0 |  | 1.1 |  | ND |  | 0.8 |  | 0.1 |  | 0.0 |  | 0.0 |  | 0.0 |  | 0.5 |  | 0.2 |  | 0.1 |  | 0.1 |
|  | NCIMB 11912^T^ |  | 0.0 |  | 0.0 |  | ND |  | 0.0 |  | ND | ≥ | 4.2 |  | 0.8 |  | 0.0 |  | 0.7 |  | 0.0 |  | 0.8 |  | 0.8 |  | 0.5 |  | 0.1 |
|  | NCIMB 11130^T^ |  | 0.0 |  | 0.0 |  | 0.2 |  | 0.0 |  | ND |  | ND |  | 0.9 |  | 0.0 |  | 0.2 |  | 0.0 |  | 0.4 |  | 1.1 |  | 0.0 |  | 1.0 |
|  | NCIMB 11129^T^ |  | 0.0 |  | 0.0 |  | 0.0 |  | 0.0 |  | 0.1 |  | 1.2 |  | 0.0 |  | 0.0 |  | 0.1 |  | 0.0 |  | 0.1 |  | 0.4 |  | 0.0 |  | 0.0 |
|  | NCIMB 11131^T^ |  | 0.0 |  | 0.3 |  | 0.0 |  | 0.1 |  | 0.6 |  | 0.0 |  | 0.0 |  | 0.7 |  | 0.0 |  | 0.0 |  | 0.0 |  | 0.1 |  | 0.0 |  | 0.0 |
|  | NCIMB 11853^T^ |  | 0.0 |  | 0.9 |  | 0.1 |  | 0.0 |  | 0.6 | ≥ | 4.0 |  | 1.3 |  | 0.0 |  | 0.0 |  | 0.1 |  | 0.0 |  | 0.4 |  | 1.5 |  | 0.9 |
|  | DSM 19304^T^ | ≥ | 3.7 |  | NT |  | 0.0 |  | NT |  | ND |  | 0.0 |  | 1.7 |  | NT |  | 1.1 |  | NT |  | 0.1 |  | 0.0 |  | 1.5 |  | 0.9 |
| Culturable Drop | DSM 18500^T^ |  | 2.0 |  | 2.6 | ≥ | 6.5 |  | 4.6 |  | 2.3 | ≥ | 6.5 |  | 3.6 |  | 3.6 |  | 1.0 |  | 0.6 |  | 0.6 |  | 0.6 |  | 0.6 |  | 1.3 |
|  | DSM 13736^T^ |  | 1.6 |  | 3.0 |  | 0.0 |  | 1.3 |  | 2.6 | ≥ | 4.5 |  | 0.3 |  | 1.0 |  | 1.0 |  | 0.5 |  | 0.8 | ≥ | 4.5 |  | 2.6 |  | 0.8 |
|  | DSM 17706^T^ |  | 0.6 |  | 1.0 |  | 1.3 |  | 1.0 |  | 2.6 | ≥ | 6.5 |  | 0.6 |  | 0.0 |  | 0.6 |  | 0.0 |  | 0.3 |  | 1.0 |  | 1.3 |  | 0.3 |
|  | DSM 15673^T^ |  | 0.0 |  | 0.0 | ≥ | 5.3 |  | 0.1 |  | 0.5 | ≥ | 5.3 |  | 0.7 |  | 0.0 |  | 0.0 |  | 0.0 |  | 0.0 |  | 0.0 |  | 0.8 |  | 0.3 |
|  | NCIMB 11912^T^ | ≥ | 1.8 |  | 0.0 |  | 1.8 |  | 0.0 |  | 1.8 | ≥ | 1.8 | ≥ | 1.8 |  | 0.0 | ≥ | 1.8 |  | 0.0 | ≥ | 1.8 | ≥ | 1.8 | ≥ | 1.8 | ≥ | 1.8 |
|  | NCIMB 11130^T^ |  | 5.7 |  | 6.4 | ≥ | 6.2 |  | 0.6 | ≥ | 6.2 | ≥ | 6.5 |  | 1.7 |  | 2.0 |  | 1.0 |  | 1.3 |  | 0.7 |  | 0.7 |  | 3.3 |  | 1.7 |
|  | NCIMB 11129^T^ |  | 1.0 |  | 1.0 |  | 4.6 |  | 0.7 |  | 3.8 | ≥ | 6.8 |  | 2.6 |  | 1.5 |  | 0.3 |  | 0.7 |  | 1.0 |  | 1.6 |  | 0.3 |  | 0.6 |
|  | NCIMB 11131^T^ |  | 2.0 |  | 1.4 |  | 0.0 |  | 2.4 |  | 4.0 |  | 2.7 |  | 2.7 |  | 1.7 |  | 1.2 |  | 0.0 |  | 1.0 |  | 0.7 |  | 1.0 |  | 0.4 |
|  | NCIMB 11853^T^ | ≥ | 3.7 |  | 0.2 |  | 0.0 | ≥ | 2.0 | ≥ | 3.7 | ≥ | 3.7 | ≥ | 3.7 |  | 4.3 |  | 0.0 |  | 0.0 |  | 0.0 |  | 0.0 |  | 1.7 |  | 1.1 |
|  | DSM 19304^T^ |  | 2.7 |  | NT | ≥ | 6.2 |  | NT | ≥ | 6.2 | ≥ | 6.5 |  | 1.7 |  | NT |  | 1.3 |  | NT |  | 5.0 |  | 1.7 |  | 1.0 |  | 1.0 |
| VBNC | DSM 18500^T^ |  | 2.0 |  | 2.6 |  | ND |  | 4.1 |  | 2.3 |  | NV |  | 3.6 |  | 3.6 |  | 1.0 |  | 0.6 |  | 0.6 |  | 0.6 |  | 0.0 |  | 1.3 |
|  | DSM 13736^T^ |  | 0.0 |  | 3.0 |  | 0.0 |  | 1.3 |  | 0.0 |  | NV |  | 0.0 |  | 1.0 |  | 0.2 |  | 0.5 |  | 0.8 |  | 0.5 |  | 0.0 |  | 0.0 |
|  | DSM 17706^T^ |  | 0.6 |  | 1.0 |  | 0.5 |  | 1.0 |  | 2.6 |  | NV |  | 0.6 |  | 0.0 |  | 0.6 |  | 0.0 |  | 0.0 |  | 1.0 |  | 1.3 |  | 0.3 |
|  | DSM 15673^T^ |  | 0.0 |  | 0.0 | ≥ | 5.3 |  | 0.0 |  | ND | ≥ | 4.4 |  | 0.6 |  | 0.0 |  | 0.0 |  | 0.0 |  | 0.0 |  | 0.0 |  | 0.6 |  | 0.1 |
|  | NCIMB 11912^T^ | ≥ | 1.8 |  | 0.0 |  | ND |  | 0.0 |  | ND |  | NV | ≥ | 1.0 |  | 0.0 | ≥ | 1.1 |  | 0.0 | ≥ | 1.1 | ≥ | 1.1 | ≥ | 1.3 | ≥ | 1.7 |
|  | NCIMB 11130^T^ |  | 5.7 |  | 6.4 | ≥ | 5.9 |  | 0.6 |  | ND |  | ND |  | 0.7 |  | 2.0 |  | 0.8 |  | 1.3 |  | 0.3 |  | 0.0 |  | 3.2 |  | 0.7 |
|  | NCIMB 11129^T^ |  | 1.0 |  | 1.0 |  | 4.6 |  | 0.7 |  | 3.7 | ≥ | 5.6 |  | 2.6 |  | 1.5 |  | 0.3 |  | 0.7 |  | 0.9 |  | 1.2 |  | 0.3 |  | 0.6 |
|  | NCIMB 11131^T^ |  | 2.0 |  | 1.2 |  | 0.0 |  | 2.3 |  | 3.4 |  | 2.7 |  | 2.7 |  | 1.1 |  | 1.2 |  | 0.0 |  | 1.0 |  | 0.7 |  | 1.0 |  | 0.4 |
|  | NCIMB 11853^T^ | ≥ | 3.7 |  | 0.0 |  | 0.0 | ≥ | 2.0 | ≥ | 3.1 |  | NV | ≥ | 2.4 |  | 4.3 |  | 0.0 |  | 0.0 |  | 0.0 |  | 0.0 |  | 0.2 |  | 0.2 |
|  | DSM 19304^T^ |  | 0.0 |  | NT | ≥ | 6.2 |  | NT |  | ND | ≥ | 6.4 |  | 0.0 |  | NT |  | 0.2 |  | NT |  | 4.9 |  | 1.6 |  | 0.0 |  | 0.1 |

^a^NT: Could not be tested; ^b^ND: Could not be determined
